# Testing Psychometric Properties of the Standard Chinese Version of the European Organization for Research and Treatment of Cancer Quality of Life Core Questionnaire 30 (EORTC QLQ-C30)

**DOI:** 10.2188/jea.14.193

**Published:** 2005-03-18

**Authors:** Hong Zhao, Katsuya Kanda

**Affiliations:** 1 School of Nursing, Peking Union Medical College.; 2 Department of Nursing Administration, Graduate School of Medicine, The University of Tokyo.

**Keywords:** Quality of Life, Questionnaires, Psychophysiologic Disorders, Neoplasms, China

## Abstract

BACKGROUND: Because cancer is now the first and second leading causes of death in both of urban and suburban area in China, there are increasing demands for measurement tools to assess quality of life in Chinese cancer patients. The objective of this study was to evaluate the psychometric properties of the standard Chinese version of the European Organization for Research and Treatment of Cancer Quality of Life Core Questionnaire 30 (EORTC QLQ-C30, version 3.0).

METHODS: The questionnaire was administered before, during, and after treatment of 143 patients with breast, gynecological, or lung cancer in six hospitals in China.

RESULTS: Cronbach’s alpha coefficients for multi-item scales were greater than 0.70 before and during treatment, except for the cognitive functioning scale. Multitrait scaling analysis showed that most of the item-scale correlation coefficients met the standards of convergent and discriminant validity. All scales and items exhibited good reproducibility, criterion-related validity, and construct validity. Score changes over time were observed in the following scales: physical, role, and social functioning; global quality of life; fatigue; nausea/vomiting. Score changes were also observed in the appetite loss item.

CONCLUSION: The standard Chinese version of the EORTC QLQ-C30 is overall a valid instrument to assess the quality of life of Chinese cancer patients.

Rapid economic development in China has resulted in changes of Chinese lifestyles and environmental conditions. Instead of infectious and chronic diseases, cancer has become the leading cause of death in China.^1^ There is an increasing public and health care professional demand for measurement tools assessing the quality of life (QOL) in Chinese cancer patients. Several cancer-specific QOL questionnaires have been developed by Chinese researchers and used in clinical trials.^2^^-^^4^ There is also a growing number of international collaborative studies for new anti-cancer drugs and treatment protocols; therefore, QOL questionnaires need cross-cultural adaptations.

The European Organization for Research and Treatment of Cancer (EORTC) Quality of Life Study Group developed the Core Questionnaire 30 (QLQ-C30) in the 1980s. The QLQ-C30 has been translated into nearly 30 languages, and its psychometric properties have been tested by several researchers in studies of patients with heterogeneous cancer types.^5^^-^^7^ One of the authors (HZ) translated the QLQ-C30 (version 2.0) into standard Chinese and evaluated a part of the psychometric properties with a group of Chinese gynecological cancer patients.^8^ The results showed that scaling success was not found in the following three items: one item in the physical functioning scale, and two items in the cognitive functioning scale. In addition, the internal consistency of the physical and cognitive functioning scales did not meet the minimal standards of Cronbach’s alpha coefficient, which is larger than 0.70.^8^ Other published studies have also reported similar limitations of the QLQ-C30 version 2.0.^5^^,^^6^^,^^9^ In the revised QLQ-C30 version 3.0, the EORTC Quality of Life Study Group modified the response categories of the physical functioning scale from dichotomous “yes/no” responses to 4-point Likert scales, ranging from “1” representing “not at all” to “4” representing “very much.” Preliminary data from the National Cancer Institute of Canada Clinical Trials Group indicated that Cronbach’s alpha coefficient was likely to be greater than 0.80 with the new QLQ-C30 version 3.0.^6^ Given such improved psychometric properties of the newer version of the QLQ-C30, the standard Chinese version of the QLQ-C30 version 2.0 was also updated to a newer version 3.0. Furthermore, the previous psychometric study of the standard Chinese version of the QLQ-C30 (version 2.0) was conducted with only Chinese gynecological cancer patients.^8^ The QLQ-C30 is intended to be used as a “core” questionnaire for patients with various types of cancer; therefore, testing its psychometric properties among patients with different types of cancer may add to the existing knowledge base regarding its clinical utility and generalizability.

The purpose of this study was to evaluate the psychometric properties and responsiveness of the standard Chinese version of the EORTC QLQ-C30 (version 3.0) with breast, gynecological and lung cancer patients in China.

## METHODS

### Patients

Because the cases of cancer, such as lung cancer, breast cancer, ovarian cancer, and carcinoma of endometrium are increasing rapidly in China, the breast cancer, gynecology cancer and lung cancer patients were recruited from six hospitals affiliated with four universities or an institute of tuberculosis and chest cancer (Table 1). These six hospitals were all appraised as the first grade hospitals by Ministry of Public Health. Patients in these hospitals came from whole country. The inclusion criteria were as follows: having a confirmed diagnosis of breast, gynecological, or lung cancer; having been scheduled to receive chemotherapy or radiation therapy on an inpatient status; being 18 years or older; having the ability to read and write Chinese; and agreeing to participate in the study. Patients who had a life expectancy of less than 6 months were excluded.

**Table 1.** The number of cases who were recruited from each hospital.

	Breast cancer	Gynecological cancer	Lung cancer
General hospital		9	5
Oncology hospital A	16	15	15
Oncology hospital B	15	8	9
Oncology hospital C	16	3	5
Maternity hospital		12	
Tuberculosis and chest cancer hospital			15

In order to evaluate the reproducibility of the standard Chinese version of the QLQ-C30, breast cancer patients were recruited from one study hospital. The eligibility criteria for such a purpose included the following: women aged 18 years or older; having the ability to read and write Chinese; having completed chemotherapy or radiation therapy; being in a stable condition; and agreeing to participate in the study.

### Questionnaires

The EORTC QLQ-C30 (version 3.0) is a 30-item questionnaire, including multi-item subscales and single items reflecting the multidimensionality of the construct of QOL. A raw score of each subscale/item in the QLQ-C30 is linearly transformed into a score, ranging from 0 to 100. The higher score of the functional subscale and the global QOL represent the higher level of functioning and QOL. The higher score of a symptom subscale or item represents the higher (more severe) symptom level.^10^

The Medical Outcomes Study 36-Item Short Form (SF-36) is a health survey questionnaire comprising 36-item questions from eight subscales. A raw score are linearly converted into a score ranging from 0 to 100. Higher scores indicate higher levels of functioning or wellbeing. The SF-36 has been translated into Chinese, and its psychometric analyses showed that the Chinese version satisfied the conventional psychometric criteria.^11^

The Karnofsky Performance Status (KPS) categorizes a patient’s activity level using an 11-point ordinal level scale, ranging from 0 “dead” to 100 “normal activities with no signs or symptoms”.^12^ This instrument has also been translated into Chinese and is widely used as a prognostic variable for cancer patients in China and worldwide.^13^

### Timing of the Data Collection and Assessment

The timing of administering the standard Chinese version of the QLQ-C30 was decided according to the regimen of therapy. The many of cancer patients are hospitalized during their “treatment period” (with multiple treatment cycles) and are discharged during the “rest period” because chemo- or radiation-therapy is generally administered in hospital settings in China. In order to reduce the risk of missing data under limited research funding and resources, the patients were requested to fill out the QLQ-C30 at the following four-time points during their hospitalization stays:

T1 (baseline): Patients completed questionnaires prior to their first cycle of treatment;

T2: Patients completed questionnaires on the last day of the first cycle of treatment;

T3: Patients completed questionnaires on the last day of the third or fourth cycle of treatment (which was dependent on their treatment schedules); and

T4: Patients completed questionnaires on the last day of the last cycle of treatment (the fourth or sixth cycle, depending on their treatment schedules).

The Chinese version of the SF-36 was administered only at T1 and T2 in an effort to decrease the respondent burden. Sociodemographic data, including age, sex, marital status, education, and occupation of the patient were collected. Clinical data including diagnosis, the cancer stage, and the type of treatment were extracted from the patients’ medical records. The KPS was rated by each patient’s nurse at the above-mentioned four time points.

To establish the reproducibility of the QLQ-C30, the recruited patients with breast cancer were further asked to complete the questionnaires twice, with a two-week interval at home. The consent forms and the questionnaires were mailed out to the breast cancer patients to be filled out.

### Statistical Analysis

Descriptive statistics were calculated to evaluate the completeness of the questionnaires and to characterize the score distributions. The internal consistency of each subscale was assessed by Cronbach’s alpha coefficient at T1 and T2 as a part of the reliability testing. Cronbach’s alpha was considered to be acceptable as a stable and internally consistent measure when it was equal to 0.70 or greater.^5^ The reproducibility of the questionnaire was evaluated by the test-retest method.

Multitrait scaling analysis^14^ was employed to test item convergent and discriminant validity. The following two criteria were used: (1) convergent validity is supported when an item-subscale correlation is 0.40 or greater; and (2) discriminant validity is supported when an item-subscale correlation is higher than correlations with other scales.

Correlation coefficients between the QLQ-C30 and the SF-36 were calculated at T1 and T2 to evaluate the criterion-related validity of the QLQ-C30. Two approaches were taken to evaluate its construct validity. The first approach involved examining the correlation coefficients among the various scales in the questionnaire at T1 and T2. It was hypothesized that conceptually related subscales would correlate substantially high with each other (Pearson’s correlation coefficient ≥ 0.40). It was considered undesirable if Pearson’s correlation coefficient between subscales was too high, such as above 0.70; such a high correlation would raise the question about the distinctiveness of the different concepts being measured by different subscales.^5^ In the second approach, the known-groups method was used to assess the clinical significance and validity.^5^ One-way analysis of variance (ANOVA) was used to test the extent to which the scores of the QLQ-C30 were able to discriminate between the subgroups of patients with different disease stages (with or without distant metastasis), kinds of cancer, and the KPS (The patients were divided into two groups, KPS score ≧ 70 group and < 70 group. According to criteria of KPS, patients whose KPS score are equal or greater than 70 can take care themselves completely).

The responsiveness of the QLQ-C30 was tested by examining how patients’ scores changed over time. The repeated measures analysis with a general linear mixed model was used to analyze the changes of the QLQ-C30 scores among three designated subgroups with different trajectories of the KPS score (i.e., The Increased KPS group: KPS_T4_-KPS_T1_ ≥ 20; the Unchanged KPS group: |KPS_T4_-KPS_T1_| < 20; the Deteriorated KPS group: KPS_T1_-KPS_T4_ ≥ 20). The KPS score group, time, and interaction of the KPS score groups by time were treated as fixed effects. In this analysis, the scores of the QLQ-C30 at baseline were used as a covariate. An F-test was conducted for each of the three fixed effects (the KPS score group, time, and interaction of the KPS groups by time), using the first-order autoregressive structure. Four different covariance structures (compound-symmetry, first-order autoregressive, variance components and “unstructured”) were compared. Then, the first-order autoregressive structure was selected, which provided the best fit for the data according to Akaike’s information criterion and Schwarz’s Bayesian criterion. This study was approved by the department of clinical study administration of hospitals and informed consent was obtained from all patients.

## RESULTS

### Patient Recruitment and Follow-up

From August 2000 through September 2001, 173 patients who met the inclusion criteria were invited to participate in the study. Twenty-six patients (15.4%) declined because the study was perceived as too burdensome (n=10), or the patients felt too ill (n=16). Patients who declined to participate in the study were significantly older than the participants (mean ages of 56.0 years vs. 50.1 years, respectively; p=0.0190). One hundred and nineteen patients completed the four times on the questionnaire. Some sample attrition was inevitable: 12 patients completed the questionnaire only three times; another 12 patients completed only one follow-up survey. Four participants who completed only the baseline questionnaire were excluded from the data analysis. The main reasons for not completing the questionnaire were as follows: feeling too ill to complete it (n=9); transferring to other hospitals (n=7); termination of the study period (n=6); administrative errors (n=3); and patient deaths (n=3).

### Sociodemographic and Clinical Characteristics

The baseline characteristics of the patients (n=143) are shown in Table 2. The patients with lung cancer (42 men and 7 women) were older and had more distance metastasis than those with breast and gynecological cancer.

**Table 2.** Patient participants’ demographic and baseline characteristics (n=143).

	Breast cancer		Gynecological cancer		Lung Cancer	
		
	n	%	n	%	n	%
Total	47	100	47	100	47	100

Age (year)						
mean	48.8		45.9		55.5	
standard deviation	11.3		11.5		10.4	
range	26-68		19-72		32-71	

Education						
compulsory	4	9	7	15	7	14
junior school	8	17	9	19	12	25
senior school	16	34	14	30	12	25
diploma	13	28	9	19	8	16
university	6	13	8	17	10	20

Occupation						
agriculture	1	2	3	6	3	6
industry	5	11	5	11	6	12
office work	17	36	23	49	16	33
student	0		3	6	0	
service	2	4	4	9	3	6
housewife	2	4	0		0	
pensioner	18	38	7	15	19	39
unemployed	0		0		1	2
other	1	2	2	4	1	2

Karnofsky Performance Status*						
<70	8	17	12	26	5	10
70+	39	83	35	75	44	90

Extent of disease						
local	25	53	14	30	7	14
local regional	16	34	20	43	18	37
distance metastasis	6	13	13	28	24	49

Treatment						
chemotherapy	12	26	8	17	36	75
chemotherapy + radiotherapy	0		0		6	12
chemotherapy + surgery	32	68	38	81	4	8
chemotherapy + radiation + surgery	0		1	2	1	2
radiotherapy + surgery	3	6	0		2	4

* The Karnofsky Performance Status (KPS) is a global assessment of a patient’s self-care and ambulation abilities. The KPS categorizes a patient’s activity level using an 11-point ordinal level scale, ranging from 0 “dead” to 100 “normal activities with no signs or symptoms.”

### Descriptive Statistics

Table 3 shows the means, standard deviations, percentages of scoring at the floor and ceiling for each subscale/item, and Cronbach’s alpha coefficients for the multi-item subscales of the QLQ-C30 at both T1 and T2. Score distributions were roughly symmetrical for the majority of the functioning subscales at both T1 and T2, except for the role and cognitive functioning subscales, which showed a negative skew, especially at T1. The distribution of the symptom subscale scores and single-item scores were also skewed.

**Table 3.** Mean scores, percentages of floor and ceiling, and Cronbach’s alpha coefficients of each subscale/item in the standard Chinese version of the EORTC QLQ-C30 among patients completed questionnaires prior to their first cycle of treatment (T1) and patients completed questionnaires on the last day of the first cycle of treatment (T2) (T1: n=143, T2: n=142).

	Item no.*	Mean (Standard Deviation)		Floor (%)		Ceiling (%)		Cronbach’s alpha coefficient^†^	
			
		T1	T2	T1	T2	T1	T2	T1	T2
Functioning scales^‡^									
Physical (PF)	1 to 5	73.8 (22.0)	67.7 (22.6)	1	2	11	6	0.80	0.82
Role (RF)	6, 7	66.0 (32.7)	55.6 (34.0)	9	15	32	20	0.87	0.91
Emotional (EF)	21 to 24	68.2 (24.9)	68.2 (25.2)	2	4	18	17	0.81	0.85
Cognitive (CF)	20, 25	74.4 (25.8)	73.6 (25.9)	4	4	11	33	0.49	0.55
Social (SF)	26, 27	60.1 (29.4)	55.5 (30.5)	6	10	18	14	0.71	0.76

Global health/QOL (QL)^‡^	29, 30	58.3 (24.0)	51.5 (22.2)	4	4	6	2	0.85	0.93

Symptom scales/items^§^									
Fatigue (FA)	10, 12, 18	42.4 (26.9)	48.3 (26.2)	9	5	5	8	0.83	0.83
Nausea and vomiting (NV)	14, 15	15.7 (23.3)	28.1 (30.3)	57	38	2	6	0.82	0.89
Pain (PA)	9, 19	28.4 (30.1)	33.9 (29.6)	34	22	4	6	0.79	0.75
Dyspnea (DY)	8	34.7 (32.1)	34.3 (29.7)	34	32	10	6	-	-
Sleep disturbance (SL)	11	40.1 (30.4)	41.1 (31.9)	25	25	8	12	-	-
Appetite loss (AP)	13	34.3 (33.1)	47.4 (32.8)	39	21	8	15	-	-
Constipation (CO)	16	25.4 (31.0)	32.2 (35.4)	52	46	6	11	-	-
Diarrhea (DI)	17	14.0 (22.2)	16.7 (26.3)	68	66	0	1	-	-
Financial impact (FI)	28	52.2 (33.7)	54.2 (33.4)	18	18	20	20	-	-

* The numerals correspond to the item numbers in the questionnaire.

^†^ Cronbach’s alpha coefficients > 0.70 indicate adequate scale internal consistency.

^‡^ Scores range from 0 to 100. The higher scores represent higher levels of functioning and global quality of life (QOL).

^§^ Scores range from 0 to 100. The higher scores represent higher levels of symptoms or problems.

### Reliability

Eight of the nine multi-item subscales met the minimal standards of reliability (Cronbach’s alpha coefficient > 0.70), but only the cognitive functioning scale did not meet this standard (Table 3).

Table 4 shows that all of the subscales/items of the QLQ-C30 exhibited good stability with Pearson’s correlation coefficients, ranging from 0.81 (constipation item) to 0.93 (emotional and global health/QOL subscales).

**Table 4.** Pearson’s correlation coefficients of test-retest among breast cancer patients (n=36).

Scales/items	correlation coefficient
physical functioning (PF)	0.88
role functioning (RF)	0.85
emotional functioning (EF)	0.93
cognitive functioning (CF)	0.90
social functioning (SF)	0.89
global health/QOL (QL)	0.93
fatigue (FA)	0.91
nausea and vomiting (NV)	0.83
pain (PA)	0.86
dyspnea (DY)	0.82
sleep disturbance (SL)	0.87
appetite loss (AP)	0.91
constipation (CO)	0.81
diarrhea (DI)	0.87
financial impact (FI)	0.88

### Validity

Table 5 shows Pearson’s correlation coefficients between each item and its own subscale at T1 and T2. The absolute value of the majority of item-subscale correlation coefficients exceeded the criterion of 0.40 for item-convergent validity at T1 and T2, with the exception of items 20 and 25 (rs = 0.33 - 0.38). The majority of items, in general, correlated higher with the subscales to which the items belong than the coefficients with the other subscales to which the items do not belong; however, there were exceptions regarding items 1, 9, 10, 20, and 25 at T1 and T2.

**Table 5.**
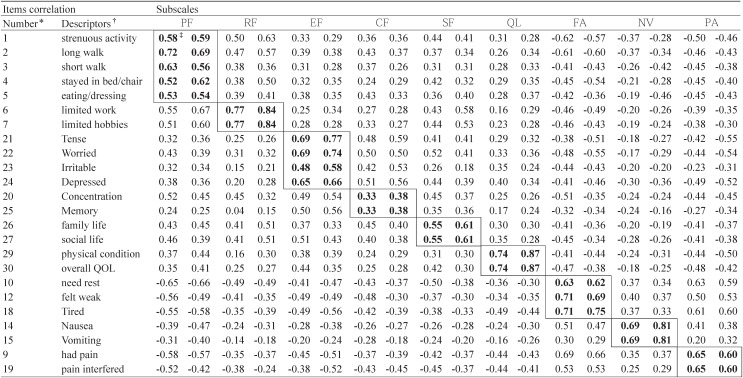
Pearson’s correlation coefficients between the items and scales in the EORTC QLQ C-30 among patients completed questionnaires prior to their first cycle of treatment (T1) and patients completed questionnaires on the last day of the first cycle of treatment (T2)

* The item number corresponds to the number of each item in the questionnaire.

^†^ The brief item descriptors approximate the questions asked in each item. Abbreviations represent: physical functioning (PF), role (RF), emotional (EF), cognitive (CF), social (SF), and global health/QOL (QL); symptom scales/items include: fatigue (FA), nausea and vomiting (NV); pain (PA); dyspnea (DY); sleep disturbance (SL); appetite loss (AP); constipation (CO); diarrhea (DI); and financial impact (FI).

^‡^ Pearson’s correlation coefficients between the subscales and items within the same subscale (corrected for overlap) are in bold lettering. Under each heading, the first column is among patients completed questionnaires prior to their first cycle of treatment (T1), and the second column is among patients completed questionnaires on the last day of the first cycle of treatment (T2).

Table 6 shows the results of criterion-relation validity. Using the SF-36 as the external standard, the correlation coefficients between the relevant subscales of the QLQ-C30 and the SF-36 were moderate (ranging from 0.38 to 0.57 at T1, and from 0.38 to 0.62 at T2).

**Table 6.** Pearson’s correlation coefficients of subscales between the Standard Chinese version of the EORTC QLQ-C30 and the Chinese version of the SF-36 among patients completed questionnaires prior to their first cycle of treatment (T1), and the second column is among patients completed questionnaires on the last day of the first cycle of treatment (T2).

Subscales of SF36	Scales of QLQ-C30																	

	PF		RF		EF		CF		SF		QL		FA		NV		PA	
Physical functioning (PF)	0.57	0.50	0.51	0.45	0.43	0.36	0.40	0.32	0.37	0.25	0.22	0.25	-0.44	-0.43	-0.23	-0.31	-0.40	-0.41
role-physical (RP)	0.34	0.35	0.38	0.42	0.24	0.29	0.35	0.25	0.30	0.34	0.28	0.31	-0.40	-0.43	-0.30	-0.22	-0.31	-0.32
bodily pain (BP)	0.41	0.29	0.35	0.10	0.32	0.31	0.33	0.23	0.40	0.25	0.29	0.32	-0.43	-0.43	-0.34	-0.20	-0.58	-0.62
general health perception (GH)	0.25	0.20	0.07	0.20	0.35	0.35	0.37	0.38	0.29	0.24	0.41	0.45	-0.35	-0.28	-0.22	-0.17	-0.27	-0.34
vitality (VT)	0.51	0.45	0.24	0.27	0.54	0.57	0.43	0.42	0.42	0.34	0.41	0.46	-0.57	-0.61	-0.33	-0.32	-0.49	-0.63
social functioning (SF)	0.42	0.39	0.32	0.42	0.31	0.41	0.29	0.32	0.49	0.59	0.29	0.38	-0.53	-0.45	-0.28	-0.30	-0.37	-0.49
role-emotional (RE)	0.22	0.26	0.39	0.38	0.39	0.31	0.28	0.36	0.21	0.35	0.24	0.28	-0.27	-0.31	-0.22	-0.23	-0.20	-0.38
mental health.(MH)	0.36	0.30	0.24	0.25	0.53	0.56	0.38	0.41	0.36	0.28	0.37	0.38	-0.32	-0.38	-0.20	-0.17	-0.34	-0.49

Pearson’s correlation coefficients of relevant subscales between the standard Chinese QLQ-C30 and the Chinese SF-36 are in underline.

Under each heading, the first column is T1, and the second column is T2.

Table 7 shows Pearson’s correlation coefficients between the subscales. The moderate inter-subscale correlation coefficients (r ≥ 0.60) were found between some conceptually related subscales, such as the physical functioning and fatigue subscales (r = -0.68). All of the inter-scale correlation coefficients were less than 0.70.

**Table 7.** Pearson’s correlation coefficients among subscales in the Standard Chinese version of the EORTC QLQ C-30 among patients completed questionnaires prior to their first cycle of treatment (T1, n=143), and the second column is among patients completed questionnaires on the last day of the first cycle of treatment (T2, n=142).

Scales	PF	RF	EF	CF	SF	QL	FA	NV	PA
Physical functioning (PF)		0.57	0.45	0.47	0.50	0.37	-0.68	-0.38	-0.61
role functioning (RF)	*0.66*		0.28	0.32	0.46	0.20	-0.48	-0.21	-0.41
emotional functioning (EF)	*0.44*	*0.32*		0.60	0.50	0.43	-0.54	-0.27	-0.46
cognitive functioning (CF)	*0.43*	*0.29*	*0.65*		0.48	0.26	-0.51	-0.30	-0.44
social functioning (SF)	*0.47*	*0.58*	*0.42*	*0.44*		0.37	-0.49	-0.27	-0.48
global health/QOL (QL)	*0.44*	*0.30*	*0.38*	*0.30*	*0.31*		-0.46	-0.22	-0.48
fatigue (FA)	*-0.67*	*-0.48*	*-0.59*	*-0.41*	*-0.39*	*-0.42*		0.44	0.67
nausea and vomiting (NV)	*-0.46*	*-0.26*	*-0.33*	*-0.24*	*-0.25*	*-0.29*	*0.40*		0.34
pain (PA)	*-0.55*	*-0.34*	*-0.58*	*-0.48*	*-0.41*	*-0.47*	*0.67*	*0.37*	

The negative values refer to an inverse correlation: e.g., the higher the fatigue, the lower the physical functioning.

Numbers above the diagonal are T1, while those below (in italics) are T2.

Using the first and second assessments of the performance status ratings as indicators of the changes in QOL, the total patients were divided into two groups: patients with a performance status score of less than 70, and patients with a performance status score equal to or greater than 70. At T1 (Table 8), significant differences (p < 0.05) were observed in the functioning subscales, the global QOL subscale, the fatigue subscale, and the items of sleep disturbance, appetite loss, and constipation. There were significant differences (p < 0.05) between the two groups in terms of the physical, role, emotional, and cognitive functioning subscales, as well as the fatigue, pain, dyspnea, sleep disturbance, and appetite loss subscales/items at T2.

**Table 8.** Differences of mean scores of subscales: Comparisons between two known-groups with the Karnofsky Performance Status score ≧70 or <70 subgroups among patients completed questionnaires prior to their first cycle of treatment (T1), and the second column is among patients completed questionnaires on the last day of the first cycle of treatment (T2).

Scales/items	T1		T2	
	
	Difference	p	Difference	p
physical functioning (PF)	-22.0	0.0116	-21.1	0.0001
role functioning (RF)	-24.2	0.0006	-15.0	0.0216
emotional functioning (EF)	-12.3	0.0236	-14.1	0.0035
cognitive functioning (CF)	-11.8	0.0386	-14.2	0.0157
social functioning (SF)	-20.4	0.0014	-8.6	0.1427
global health/QOL (QL)	-10.5	0.0467	-6.1	0.1560
fatigue (FA)	23.5	0.0000	18.0	0.0020
nausea and vomiting (NV)	11.6	0.0793	12.7	0.0640
pain (PA)	17.2	0.0526	20.3	0.0033
dyspnea (DY)	11.2	0.2098	14.9	0.0090
sleep disturbance (SL)	14.4	0.0310	16.9	0.0056
appetite loss (AP)	23.1	0.0013	20.8	0.0008
constipation (CO)	17.8	0.0087	9.0	0.1875
diarrhea (DI)	-2.4	0.6237	-6.2	0.2220
financial impact (FI)	9.4	0.2046	4.3	0.5096

Among the KPS subgroups in each cancer group, the results of breast cancer were more similar with the results of entire group (The data are not shown).

Among the subgroups by disease stage (i.e., local only, local regional, and distance metastasis), significant differences were observed in the physical and role functioning subscales (p < 0.05) at T1. There were no significant differences in terms of any other subscales/items at T2.

Among the disease stage subgroups in each cancer group, significant difference was observed in pain subscale at T1 in lung cancer group (The data are not shown).

Among subgroups by cancers, significant differences were observed in the physical functioning subscale, role functioning subscale at both T1 and T2, as well as in fatigue subscale at T2 (Table 9).

**Table 9.** Difference of Mean score among primary cancer among patients completed questionnaires prior to their first cycle of treatment (T1), and the second column is among patients completed questionnaires on the last day of the first cycle of treatment (T2).

Scales/items	Breast cancer mean ± standard deviation		Gynecology cancer mean ± standard deviation		Lung cancer mean ± standard deviation		p	
PF	68.9±23.1	80.7± 9.5	61.4±25.7	69.7±24.7	72.6±17.3	71.0±20.2	0.047	0.028
RF	62.7±32.4	81.9±24.3	44.0±36.7	50.4±32.7	60.2±30.2	65.6±32.9	0.014	<0.001
EF	69.2±22.2	70.0±22.4	63.5±28.9	65.6±26.8	71.8±24.0	68.9±25.5	0.267	0.671
CF	70.7±24.9	77.7±23.1	73.4±29.0	69.1±28.0	76.5±23.8	76.2±25.9	0.545	0.233
SF	54.3±30.3	61.7±28.6	49.3±31.1	55.3±32.2	62.6±29.2	63.3±27.2	0.097	0.380
QL	52.0±23.6	59.8±25.4	46.6±24.4	59.2±23.3	55.8±17.5	56.1±23.6	0.127	0.728
FA	43.0±25.7	34.2±23.6	54.8±28.2	50.4±26.9	46.9±23.8	42.6±28.2	0.084	0.013
NV	27.2±26.6	15.2±24.5	34.4±34.3	16.0±20.5	22.8±28.8	16.0±25.0	0.167	0.985
PA	30.1±29.7	23.8±28.4	41.8±31.6	30.5±31.3	29.9±26.3	31.0±30.4	0.080	0.430
DY	31.9±28.9	26.2±28.6	37.6±34.5	36.9±34.2	33.3±25.5	40.8±32.1	0.630	0.071
SL	37.7±25.0	35.5±27.6	49.6±37.3	44.7±29.7	36.1±31.1	40.1±33.3	0.077	0.349
AP	46.4±33.3	27.0±30.8	53.9±32.3	40.4±32.6	42.2±34.5	35.4±35.0	0.210	0.137
CO	35.5±34.0	32.6±32.2	31.9±37.4	24.8±29.0	29.3±35.1	18.8±30.7	0.692	0.091
DI	16.7±24.1	14.9±21.8	16.3±27.7	10.6±18.5	17.0±27.3	16.3±25.6	0.992	0.431
FI	55.8±31.5	52.5±27.6	59.6±32.6	57.4±39.1	47.6±35.4	46.9±33.3	0.200	0.313

Under each heading, the first column is T1, and the second column is T2.

Abbreviations represent: physical functioning (PF), role (RF), emotional (EF), cognitive (CF), social (SF), and global health/QOL (QL); symptom scales/items include: fatigue (FA), nausea and vomiting (NV), and pain (PA), dyspnea (DY), sleep disturbance (SL), appetite loss (AP), constipation (CO), diarrhea (DI), and financial impact (FI).

A repeated-measures ANOVA adjusted for the scores at T1 showed significant time effects in the physical (F = 11.74, p = 0.0001), role (F = 13.42, p = 0.0001) and social (F = 6.44, p = 0.0003) functioning subscales, the global QOL (F = 4.52, p = 0.0410), the fatigue subscale (F = 6.37, p = 0.0003), the nausea/vomiting subscale (F = 15.27, p = 0.0001), and the appetite loss item (F = 9.31, p = 0.0001). Significant effects of the KPS score groups were observed in the social functioning subscale (F = 4.80, p = 0.0086), the global QOL subscale (F = 3.25, p = 0.0396), the fatigue subscale (F = 4.80, p = 0.0086) and the appetite loss item (F = 3.26, p = 0.0393). Significant interactions of the KPS score groups by time were observed in the social functioning subscale (F = 2.30, p = 0.0349), and the nausea/vomiting subscale (F = 2.31, p = 0.0334).

## DISCUSSION

Previous studies of the EORTC QLQ-C30 have shown that it is a valid and reliable scale that is sensitive enough to respond to the changes of cancer patients’ conditions across various countries.^5^^-^^7^^,^^9^ In this study, the psychometric properties of the standard Chinese version of the QLQ-C30 (version 3.0) were evaluated in a repeated measure design study with lung, gynecological, and breast cancer patients. The study yielded results that generally satisfied the conventional psychometric criteria before and during treatment (T1 and T2).

The descriptive statistics showed ceiling effects in some items of the physical functioning scale and floor effects in the symptom scales/items before treatment (T1). However, the floor and ceiling effects at T2 were smaller than those effects at T1. This result may be attributed to the impact of chemo- or radiation therapy on patients’ QOL; such anti-cancer therapy, in general, is likely to bring about decreased patients’ physical and mental functioning and increased side effects of anti-cancer drugs or radiation.

Of the nine subscales in the standard Chinese QLQ-C30, Cronbach’s alpha coefficients for eight subscales were above 0.70. Multitrait scaling analysis showed that most of the item-subscale correlation coefficients met the standards of convergent and discriminant validity. It is worth noting that Cronbach’s alpha coefficients of the physical functioning subscale at both T1 and T2 were greater than 0.70. Such a result is consistent with the preliminary data from the National Cancer Institute of Canada Clinical Trials Group.^6^ All of the subscales and items exhibited excellent reproducibility, with correlation coefficients ranging from 0.81 to 0.93. The correlation coefficients between the relevant subscales of the QLQ-C30 and the SF-36 indicated good criterion-related validity. All inter-subscale correlation coefficients, which were less than 0.70 at both T1 and T2, indicated that each subscale measured a unique concept in relation to the other subscales.

Similar to the previous study’s findings,^8^ Cronbach’s alpha coefficients and the results of multitrait scaling analysis of the cognitive functioning subscale were in question. Cronbach’s alpha coefficients of the cognitive functioning subscale at T1 and T2 were less than 0.40. The item-subscale correlation coefficients of items 20 and 25 with the cognitive functioning subscale were smaller than those coefficients with the emotional functioning subscale (at T1, r = 0.49 and 0.50, respectively; at T2, r = 0.54 and 0.56, respectively) and with the other subscales (e.g., the social and role functioning subscales). Despite the notion that memory and concentration appear to be two distinctive aspects of cognitive functioning, a single composite index of the overall cognitive functioning scale would probably have general clinical utility.

Although Cronbach’s alpha coefficients of the physical functioning subscale in this study were greater than 0.70, a correlation coefficient between item 1 (strenuous activity) and the physical functioning subscale was less than that with the other scales (e.g., the fatigue and role functioning subscales) at both T1 and T2. This finding is also similar to the previous study.^8^ In addition, this finding seems to be attributed to the difference between the Chinese and the other cultures in terms of how to interpret the item phrase “strenuous activity.” In Chinese culture, strenuous activities for individuals with illness are socially unacceptable; they are hardly supposed to do such strenuous activities as “carrying a heavy shopping bag or a suitcase” when they are ill. The data of this study (not shown in the tables) indicated that the majority of the patients lived with their spouses, parents, or children, and received informal care from these family members. Unlike patients in American and European countries, most of the Chinese patients took sick leaves after being diagnosed and throughout the entire period of treatment. They even received support from family members or relatives with respect to house-keeping, childcare, or shopping. For such patients in Chinese culture, “strenuous activities,” strains, or stressful events appear to be more of role functioning problems or fatigue problems rather than simple physical functioning problems. In the study about Korean version of EORTC QLQ-C30 (version 3.0), all subscales met the criteria.^15^ In addition, scaling success was not found in relation to item 10 (need rest) in the fatigue subscale and item 9 (had pain) in the pain subscale. The possible reasons for such results need further investigation.

The construct validity of the QLQ-C30 was tested against patient subgroups with different clinical status. The majority of the functioning scales and the symptom subscales/items were able to clearly distinguish between patient subgroups by the KPS score (< 70 vs. ≥ 70) at both T1 and T2. The lack of statistically significant differences among the different subgroups by kinds of cancer and disease stage may suggest that, in this given sample of patients, kinds of cancer and disease stage may not be particularly useful predictors for the current levels of functioning or symptoms.

Missing data are traditionally a very serious problem in repeated measure design studies because missing data may cause statistical bias and skew the interpretation of the study results. In order to decrease the risk of missing data, multiple efforts were made by the investigator: for example, patients who had a life expectancy of more than 6 months were invited to participate in the study; patients were asked to complete the questionnaires at their hospitals; research assistant nurses were trained to prevent administrative errors. Eighty one percent (n=119) of the patients completed all three follow-up surveys. The primary reasons for participant loss during the course of the follow-up surveys were generally related to severely ill conditions and transferals to other hospitals. Because the amount of the missing data was limited, repeated-measure analysis of variance was employed in this study. Changes over time were observed in the physical, role and social functioning subscales, the global QOL subscale, the fatigue subscale, the nausea/vomiting subscale, and the appetite-loss item. Several functioning subscales and symptom subscales/items of the deteriorated KPS subgroup showed a more steady and protracted decline than did the increased KPS subgroup. Compared to the other two subgroups, the unchanged KPS subgroup had fewer steep changes in the subscale or item scores.

The following limitations should be considered while interpreting the study results. First, relatively small sample sizes of different cancer-type subgroups precluded separate subgroup analysis. Second, limited research resources (i.e., the investigator’s and research assistants’ time availability, study funding, etc.) precluded long-term follow-up of cancer survivors. Finally, the anti-cancer drugs’ delayed onset of side effects might potentially be missed through this study design. The follow-up data collection was limited to immediately after the last cycle of treatment at the study hospitals in an effort to decrease the risk of missing data.

The psychometric properties of the standard Chinese version of the EORTC QLQ-C30 (version 3.0) were tested. The results indicate that, overall, it is a valid instrument to assess the quality of life of Chinese patients with breast, gynecological, or lung cancer undergoing chemotherapy or radiation therapy. Additionally, known-group and repeated-measure analysis provided promising results regarding its clinical validity.
